# Microbiological evaluation of different reprocessing methods for cuffed and un-cuffed tracheostomy tubes in home-care and hospital setting

**DOI:** 10.3205/dgkh000262

**Published:** 2016-02-16

**Authors:** Matthias Leonhard, Ojan Assadian, Michaela Zumtobel, Berit Schneider-Stickler

**Affiliations:** 1 Department of Otorhinolaryngology, Medical University of Vienna, Austria; 2 Institute for Skin Integrity and Infection Prevention, University of Huddersfield, United Kingdom

**Keywords:** disinfection, dishwasher, ultrasound, sonification, cuff, reprocessing, tracheostomy tube, biofilm, infection control, medical device

## Abstract

**Background:** Manufacturers’ recommendations on cleaning of tracheostomy tubes focus on general warning information and non-specific manual cleaning procedures. The aim of this experimental study was to evaluate different reprocessing methods and to determine the mechanical integrity and functionality of tracheostomy tubes following reprocessing.

**Methods:** Sixteen cuffed or un-cuffed tracheostomy tubes obtained from hospital in-patients were reprocessed using one of the following reprocessing methods: a) manual brushing and rinsing with tap water, b) manual brushing followed by disinfection with a glutaraldehyde solution, c) manual brushing followed machine-based cleaning in a dishwasher, and d) manual brushing followed by ultrasound cleaning in a commercially available ultrasound device. Microbial burden of the tubes before and after reprocessing was assessed by measurement of microbial colony-forming units per mL (CFU/mL) of rinsing fluid. After cleaning, tracheostomy tubes were investigated for loss of functionality.

**Findings:** Manual brushing and rinsing with tap water reduced microbial colonization in average by 10^2^ CFU/mL, but with poor reproducibility and reliability. Complete microbial reduction was achieved only with additional chemical or machine-based thermal disinfection. Ultrasound sonification yielded no further microbial reduction after manual brushing.

**Conclusion:** Manual brushing alone will not result in complete eradication of microorganism colonising cuffed or un-cuffed tracheostomy tubes. However, manual cleaning followed by chemical or thermal disinfection may be regarded as safe and reproducible reprocessing method. If a machine-based reprocessing method is used for cuffed tubes, the cuffs’ ventilation hose must be secured in a safe position prior to thermal disinfection.

## Zusammenfassung

**Hintergrund:** Herstellerangaben zur Aufbereitung von Tracheostomiekanülen beziehen sich häufig auf generelle Warnhinweise und nicht auf spezifische Angaben zur Reinigung. Ziel der vorliegenden experimentellen Arbeit war es, unterschiedliche Aufbereitungsverfahren hinsichtlich ihrer Effektivität zu untersuchen und die mechanische Integrität von Tracheostomiekanülen nach entsprechender Aufbereitung zu beurteilen.

**Methoden:** Sechzehn geblockte oder nicht-geblockte Tracheostomiekanülen, die von stationär aufgenommenen Patienten nach Verwendung bezogen wurden, wurden folgenden Aufbereitungsmethoden zugeführt: a) manuelles Bürsten und Spülen mit Leitungswasser, b) manuelles Bürsten und anschließende Tauchdesinfektion mittels Glutaraldehydlösung, c) manuelles Bürsten mit anschließender Aufbereitung in einem Geschirrspüler und d) manuelles Bürsten mit anschließender Ultraschall(US)-Behandlung in einem kommerziell erhältlichen US-Wannengerät. Die mikrobielle Last der Kanülen wurde vor und nach Aufbereitung mittels Bestimmung der Anzahl Kolonie-bildender Einheiten pro ml Spülflüssigkeit (KbE/ml) bestimmt. Nach der jeweiligen Aufbereitung wurden die Kanülen auf funktionelle Integrität überprüft.

**Ergebnisse:** Manuelles Bürsten und Spülen mit Leitungswasser reduzierte die mikrobielle Last im Durchschnitt um 10^2^ KbE/ml, jedoch mit unzureichender Reproduzierbarkeit und Zuverlässigkeit. Eine vollständige Reduktion wurde lediglich mittels zusätzlicher chemischer oder thermisch-maschineller Aufbereitung erzielt. Eine Behandlung in einem US-Bad erbrachte nach manuellem Bürsten keine wesentliche weitere Reduktion.

**Fazit:** Manuelles Bürsten allein erzielt keine komplette Elimination von Mikroorganismen an geblockten oder nicht geblockten Tracheostomiekanülen. Manuelles Bürsten mit anschließender chemischer oder thermisch-maschineller Behandlung kann als sichere und reproduzierbare Aufbereitungsmethode angesehen werden. Wenn ein maschinelles Verfahren zur Aufbereitung geblockter Kanülen gewählt wird, müssen die Blockansatzstücke an einer sicheren Position in der Maschine verortet werden, damit diese nicht beschädigt werden.

## Introduction

During the past decades, advances in material and functionality of tracheostomy tubes have allowed improved patient care [1]. Cuffed tracheostomy tubes reduce the risk of ventilator-associated pneumonia by preventing aspiration during mechanical long-term ventilation [2]. Soft un-cuffed tracheostomy tubes made of silicone or various polymers reduce laryngeal complications of mechanical pressure, and permit patients, who still require a secured airway, to proceed with rehabilitation as out-patients or to be discharged to home-care. These benefits, however, come with a limited wear-time of polymer tracheostomy tubes, since the material is rapidly coated with a thick microbial biofilm within a few days [3], which then eventually destroys the soft functional elements of tracheostomy tubes within weeks.

Therefore, depending on the manufacturer’s recommendations on maintenance and wear time, cuffed tracheostomy tubes usually may need to be replaced after 3–7 days. Un-cuffed tubes may have a maximum wear time of less than 4 weeks. Polymer tracheostomy tubes are single-patients devices; cuffed tracheostomy tubes are frequently marketed as single-use medical devices by the manufacturer, however, un-cuffed tracheostomy tubes may need to be cleaned daily by the patients themselves or by healthcare workers in order to prevent biofilm formation and to maintain the tube’s functionality.

Manufacturers’ recommendations for un-cuffed tracheostomy tubes frequently derive from general cleaning guidelines for parts of respiratory circuits, but have not thoroughly been validated for individual tracheostomy tubes. The recommended cleaning and maintenance methods usually comprise manual brushing and rinsing of the disassembled tube parts under tap water followed by disinfection using antimicrobial compounds such as glutaraldehydes, and have been adopted by nursing best practice statements [4]. For cuffed tracheotomy tubes, specific information on cleaning and maintenance is even vaguer due to the mechanical vulnerability of the thin cuff material (Table 1 (Tab. 1)). Yet, with a validated decontamination procedure and under regular control of cuff functionality, the use of cuffed tracheostomy tubes should be possible even up to 28 days maximum device-lifetime, as proposed by several manufacturers. However, most manufacturers provide only general information including the recommendation to avoid temperatures above 65°C and mechanical stress, and to avoid the use of aggressive detergents or disinfectants capable of extracting polymer plasticizers.

Aside of physical and chemical considerations, reprocessing of tracheostomy tubes in hospital settings should be time- and cost-effective, and must conform to quality management policies. Compared to conditions at home care, hospital settings require short reprocessing hands-on times, complete quality control and strict infection control assurance in order to prevent transmission of pathogens from still unprocessed to already processed tracheostomy tubes during reprocessing. These practical requirements are met best by adoption of automated cleaning and disinfection processes, which have to be implemented and validated with specific attention to polymer material sensitivity. 

Therefore, the aim of this experimental study was to evaluate the antimicrobial efficacy of manual brushing and flushing on worn tracheostomy tubes alone, and the added effect of either disinfection using a glutaraldehyde solution, a thermal disinfection using a dishwasher, or ultrasound cleaning in a commercially available ultrasound cleaning device. The mechanical integrity and functionality of tracheostomy tubes following various reprocessing methods was also assessed.

## Methods

**Figure 1:**
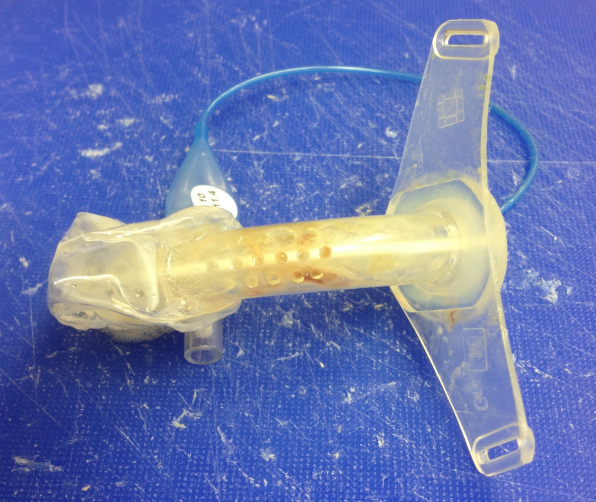
Freshly removed cuffed tracheostomy tube (Heimomed Prima-Phon II, size 8) after 72 hours of wear

Sixteen polymer tracheostomy tubes (8 un-cuffed and 8 cuffed, by Heimomed^®^, Kerpen, Germany and Teleflex Medical^®^, Kernen, Germany) were obtained from hospitalised in-patients directly after use. Un-cuffed tubes were changed and collected after minimum of 1 day of use, cuffed tubes after a minimum of 3 days of use (Figure 1 (Fig. 1)). All tubes were tested before and after manual cleaning, and were assigned to three different additional cleaning procedures following their consecutive random collection order.

### Assessment of microbial bio-burden 

Microbial concentration on the inner surface of the tubes was assessed before and after manual cleaning. The tube’s lumen was rinsed with 10 mL of sterile 0.9% saline solution (B. Braun, Melsungen, Germany). For reprocessing methods B and C (below), instead of 0.9% saline solution validated neutralizers were used. Microbial concentration of the rinsing solution was determined by using the standard microbiological serial dilution method and plating on different culture agars: Columbia 5% agar, Columbia CNA agar (selective to Gram-positive bacteria), McConkey agar (selective for Gram-negative bacteria), and Sabouraud agar (selective for yeast). These agars allow identification of a broad microbiological spectrum, which is frequently encountered in oral cavities and the respiratory tract. Colony-forming units (CFUs) were counted and morphologically identified after 24 h incubation at 37°C. 

### Investigated reprocessing procedures

The effectiveness of the applied reprocessing procedures in terms of microbial reduction was tested. This study examined four different reprocessing methods: 

**Method A** – Manual cleaning. Manual brushing with a new tube brush followed by rinsing with tap water until a macroscopically clean result was achieved.

**Method B** – Manual pre-cleaning and additional chemical disinfection: Manual pre-cleaning and complete submersion of the cleansed tubes in a commercially available and by the manufacturer recommended disinfection solution (PRIMASTOM^®^ with 2% glutaraldehyde, preparation according to manufacturer’s instruction, Heimomed^®^, Kerpen, Germany) for 1 hour at room temperature, followed by final rinsing with tap water.

**Method C** – Manual pre-cleaning followed by a machine-based thermal disinfection in a dishwasher: After manual cleaning, tubes were placed vertically in a dishwasher (MIELE G601 SC^®^, Miele^®^, Gütersloh, Germany), peak process temperature: 65° C, standard washing program (48 min) without additional cleaning agents.

**Method D** – Manual pre-cleaning followed by ultrasound sonification: Ultrasound cleaning in a commercially available ultrasound household device for cleaning jewellery and optical instruments (Ultrasonic, Fa. DEMA, Hägendorf, Switzerland, US-frequency: 42 kHz, recommended procedure by manufacturer: 6 min sonification, only water as sonic medium), final rinsing with tap water. 

### Evaluation of the material integrity after processing

Cleaned and reprocessed tracheostomy tubes were examined for visible signs of material alteration including colour changes, deformation, or other causes for loss of tube function. Cuff integrity was tested by inflation at 25 mmHg of air and documentation of the maintenance of pressure during 20 minutes with a cuff pressure manometer (Hi-Lo cuff pressure manometer, Covidien, Dublin, Ireland).

## Results

*Staphylococcus aureus*, *Staphylococcus epidermidis*, *Pseudomonas aeruginosa* and *Candida *spp. were identified as most frequent pathogens colonising the inner lumen of tracheostomy tubes. The microbial burden of the rinsing solution before cleaning ranged between 10^1^ CFU/mL and 10^6^ CFU/mL, mean: 9×10^4^ CFU/mL. Neither quality nor quantity of the microbial colonisation differed between un-cuffed and cuffed tracheostomy tubes. 

### Performance of investigated cleaning methods

In all 16 tested tubes, manual brushing achieved microbial reduction ranging between 10^1^ and 10^4^ CFU/mL, with a mean microbial reduction of 2 log_10_. However, the reliability and reproducibility of the cleaning efficiency was poor. Only manual cleaning with additional disinfection in a glutaraldehyde-based solution achieved a complete microbial elimination in all tested samples. Additional machine based thermal cleaning using a dishwasher reduced the microbial burden to less than 10^1^ CFU/mL, however, starting from an already initial low microbial bio-burden after manual pre-cleaning. Surprisingly, ultrasound sonification using a household ultrasound-device following a final rinse with tap water did not improve the results of a previously conducted manual pre-cleaning. Detailed results of the microbial colonisation of polymer tracheostomy tubes before and after application of the investigated reprocessing methods are summarised in Table 2 (Tab. 2).

### Material integrity of processed tubes

No signs of material alteration were found on any of the tested tracheostomy tubes. Cuff functionality after the single reprocessing regiments remained intact in all but one cuffed tubes. In this instance, improper fixation in the machine resulted in cutting off the cuff’s ventilation hose by a dishwasher rotor arm. 

## Discussion

**Figure 2:**
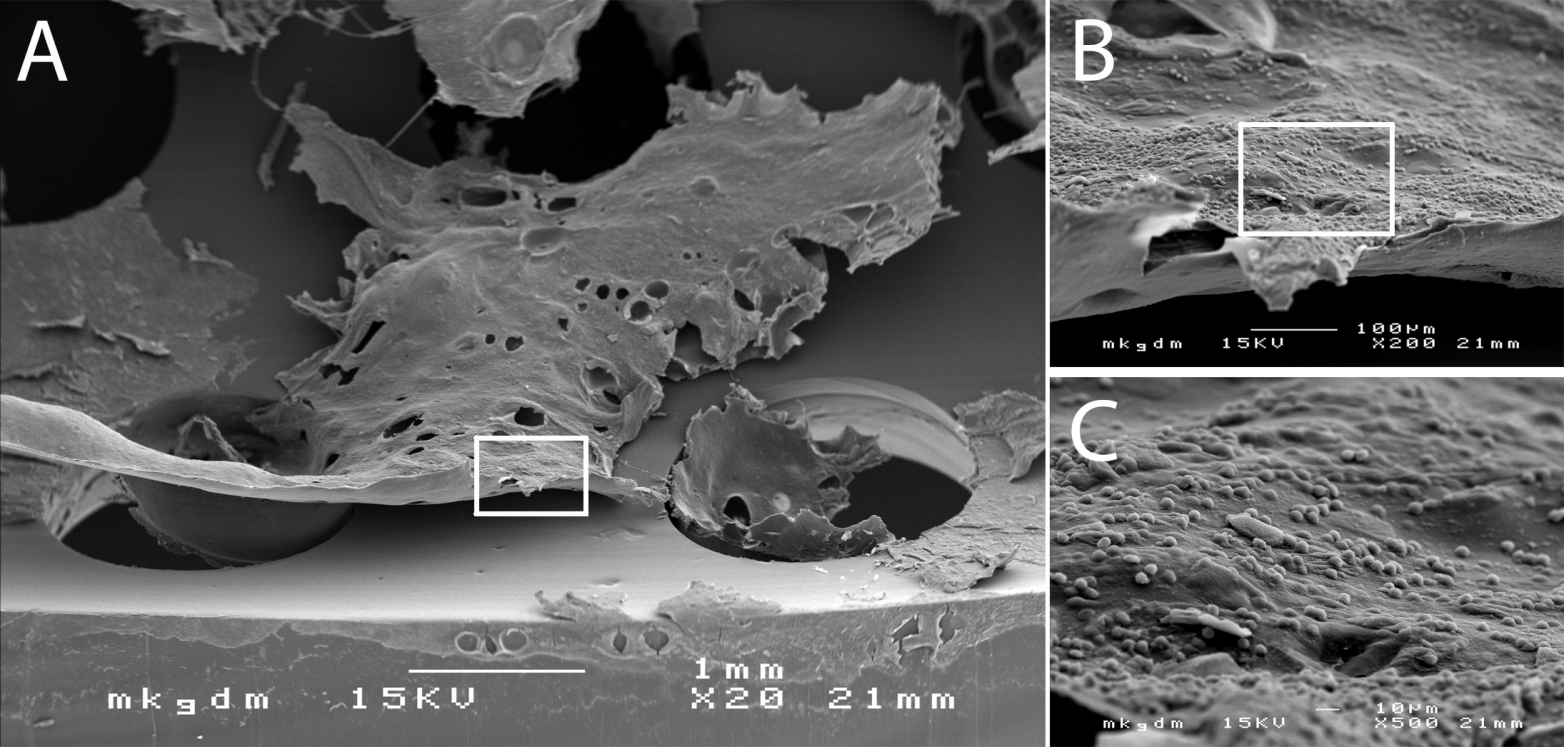
Scanning electron micrography of biofilm deposits on the inner tube surface (A). The biofilm deposits are composed of tracheal mucus with extrapolysaccaride matrix (EPS) and embedded microorganisms colonizing the trachea (B+C).

Tracheostomy tubes are semi-critical A single-patient medical devices, which allow securing the patient’s airway, and, depending of the functionality, may also assist the patient’s phonetic capability. However, because of accumulation of large deposits of mucus and debris, as well as microbial colonisation and consecutive formation of biofilm (Figure 2 (Fig. 2)), tracheostomy tubes need to be cleaned and eventually disinfected on a regular and validated basis [5].

Indeed, despite poor quality and low level of detail, reprocessing of tracheostomy tubes is briefly highlighted in most manufacturers’ instructions of use (Table 1 (Tab. 1)), and an integral part of guidelines and standards of care for cuffed or un-cuffed tracheostomy tubes [6], [7], [8], [9], [10], [11]. However, because of the increasing number of tracheostomy tube models on the market, it is difficult for scientific societies or organisations to give detailed instructions on reprocessing for all available tube models [7], [10], [11]. Therefore, it is not surprising that, although widely used, still no standardized cleaning procedures or management policies on use and reuse of tracheostomy tubes exist [2], [4], [12]. Since healthcare workers and outpatients have to follow cleaning and reprocessing instructions provided by manufacturers in order to maintain product liability, we believe that according to the European Medical Device Directive (MDD) it must be the manufacturer’s sole responsibility to provide adequate, feasible and detailed validated instructions for the care and maintenance of his medical devices. These instructions shall specifically contain detailed information on the frequency of reprocessing, the method of cleaning the tube, and information if a disinfection step is required or not. Finally, these instructions should not contain only a list of procedures and chemical compounds, which must not be applied to the tube. The whole procedure should be feasible for healthcare as well as homecare settings, and shall contain unequivocal and reproducible instructions on how to perform the complete reprocessing cycle. 

In this study, we investigated the microbial colonisation of tracheostomy tubes after 1 to 3 days of wear, and explored the microbial reduction capacity of a manual cleaning method such as manual brushing and rinsing with tap water, and the additional effect of glutaraldehyde-based disinfection, cleaning and thermal disinfection in a dishwasher, and sonification by use of an ultrasound device. With particular regard to homecare, only easily obtainable and commercially available procedures and devices were included in the study. With exception of the ultrasound sonification device, all used brushes and disinfectants are frequently available from the tube manufacturers themselves. Although the used thermal dishwasher was a medical-grade professional device, today, most household dishwashers also provide programs at 65°C, hence, allowing repeating the investigated procedure at home.

The results of this study demonstrated that manual cleaning with a tube brush and rinsing with tap water resulted in poor reliability and low reproducibility of microbial reduction. Even with good manual dexterity and sufficient time, only an average of 2 log_10_ microbial reduction was achieved. This level of reduction may be too low to prevent long-term damage to the tube caused by *Candida albicans* colonisation or in situations where the patients’ nasopharyngeal region is colonised by potentially pathogenic organisms, such as methicillin-resistant *Staphylococcus aureus* (MRSA) strains. Hence, brushing and flushing tubes with water alone seems not to be a reprocessing method, which may be recommended, even not for common household use. This procedure needs to be augmented by additional support. Indeed, Björling et al. [12] have demonstrated that already the use of a detergent during manual processing is sufficient to achieve better microbial reduction on inner cannulas of tracheostomy tubes. However, the authors also have noticed the time consuming handling of the procedure and therefore its inadequacy for hospital settings. 

Manual cleaning together with complete immersion of the pre-cleaned tube in a glutaraldehyde-based disinfection solution always achieved constant results with almost complete elimination of microbial colonisation. Considering the broad and highly reliable antimicrobial efficacy of glutaraldehydes [13], this finding is of no great surprise. However, due to the compounds potential for skin irritation and contact dermatitis [14], the use of glutaraldehyde always requires the use of adequate personal protective equipment and sufficient air ventilation in rooms, where it is used as liquid disinfectant for complete submersion of medical devices. Therefore, the compound may be used safest as antimicrobial additive for machine-based chemo-thermal disinfection processes, which is feasible in the hospital setting. Yet, uncontrolled and without adequate training, its use must be regarded as barely acceptable for household settings.

Cleaning and thermal disinfection in a dishwasher seems to be an elegant and automated alternative, which is more environmental friend than chemical disinfection. Only in one instance, less than 10 CFUs were found on the inside of a tracheostomy tube after withdrawal from the dishwasher (Tube # 10, Table 2 (Tab. 2)). Incidentally, this was the only tube where the ventilation hose of the cuff was cut off by the dishwasher rotor arm due to displacement of the device during the machine-based reprocessing process. The tube’s horizontal positioning, which prevented sufficient water flow through the tube’s inside lumen, may also explain the unchanged number of CFUs after manual pre-cleansing and consecutive reprocessing in the machine. This incident, however, undermines that if tracheostomy tubes are reprocessed in a dishwasher, a secure fixation in a vertical position is essential for both, the result of microbial reduction and prevention of the device’s integrity. Other than this incident, no detrimental impact on the material of two different tracheostomy tube models was observed in this study at a peak process temperature of 65°C. The material did not change colour or transparency, and cuff function was maintained after reprocessing. Based on these observations, tube reprocessing by use of a dishwasher at 65°C would seem to be an optimal method for regular cleansing and disinfecting tracheostomy tubes. However, the design of investigating the effect of a dishwasher on reprocessing tracheostomy tubes has several limitations, which prevent a final and well-affirmed recommendation. First, we did not investigate the microbial reduction, which can be achieved by using a dishwasher alone. The machine-based reprocessing was used only as a supportive measure after a manual brushing and flushing step. While it is likely that reprocessing tracheostomy tubes in a dishwasher alone immediately after wear would have yielded similar results as the combined manual/machine-based cycle, this remains speculative and would need to be investigated in further studies. Second, our results pertain only to few tracheostomy tube models and are difficult to be translated to other models and brands. Most importantly, however, our study is chiefly limited by the fact that all tubes underwent only one single reprocessing step. Based on the present experimental design no statement can be made on the maximum number of cycles for which the respective tracheostomy tubes can be reprocessed without being detrimentally altered in their material or their functionality. 

Ultrasound cleaning is widely used for various cleaning tasks in industry or the household setting, and may be used for pre-cleaning of dental and surgical instruments. Sonification is reported to achieve superior cleaning results compared to manual brushing, yet optimum results depend heavily on the applied frequency and the sonification medium [15], [16]. Small and affordable devices for household purposes, as used in this study, may be insufficient and cannot be recommended for effective removal of bio-burden from tracheostomy tubes. Another disadvantage of ultrasound sonification devices, regardless of their build-type, their kHz capacity, or the potential possibility to use an antimicrobial disinfection solution as sonification medium is the fact that at least a manual pre-flushing with water is required in order to remove large deposits of mucus and organic debris. 

In conclusion, the results of this study demonstrate that manual pre-cleaning alone will not result in complete eradication of microorganism colonising tracheostomy tubes. Manual cleaning followed by chemical or thermal disinfection may be regarded as safe and reproducible reprocessing method for cuffed and uncuffed tracheostomy tubes. If a machine-based reprocessing method is used for cuffed tubes, the cuffs’ ventilation hose must be secured in a safe position prior to thermal disinfection in a dishwasher.

## Notes

### Competing interests

The authors declare that they have no competing interests.

### Acknowledgements

This study was supported by the Jubilee Fund (Grant No.: AP1117) of the Austrian National Bank. The authors wish to thank Mrs Maria Stadler for excellent laboratory support. 

## Figures and Tables

**Table 1:**
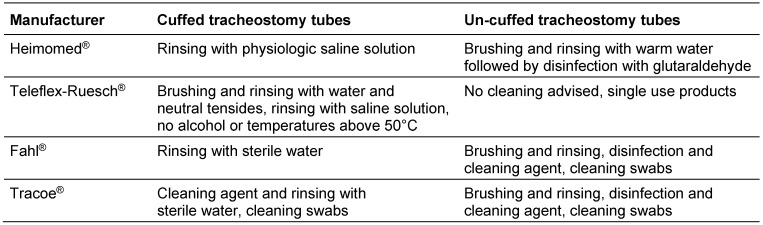
Summary of cleaning procedures as recommended by selected tube manufacturers

**Table 2:**
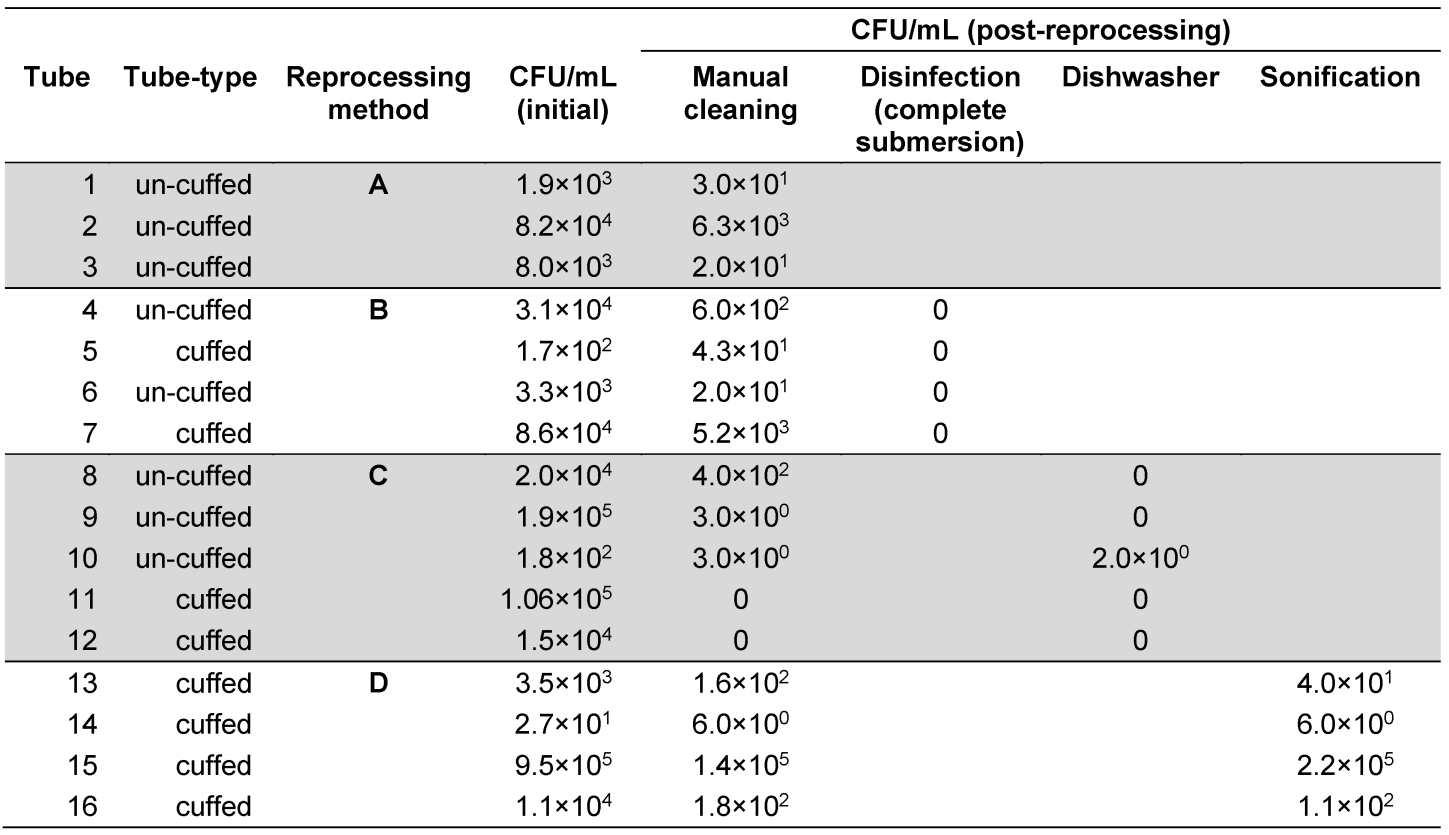
Microbial colonisation of polymer tracheostomy tubes before and after appliance of selected cleaning methods (colony forming units per ml)
